# Supplementary material for: Non-homogenous intratumor ionizing radiation doses synergize with PD1 and CXCR2 blockade
Source: Nat Commun. 2024 Oct 14;15:8845. doi: 10.1038/s41467-024-53015-9 (PMC11471822; doi:10.1038/s41467-024-53015-9)
Supplement: Supplementary file 1 — Supplementary Information [file 41467_2024_53015_MOESM1_ESM.pdf]

Supplemental Figure 1

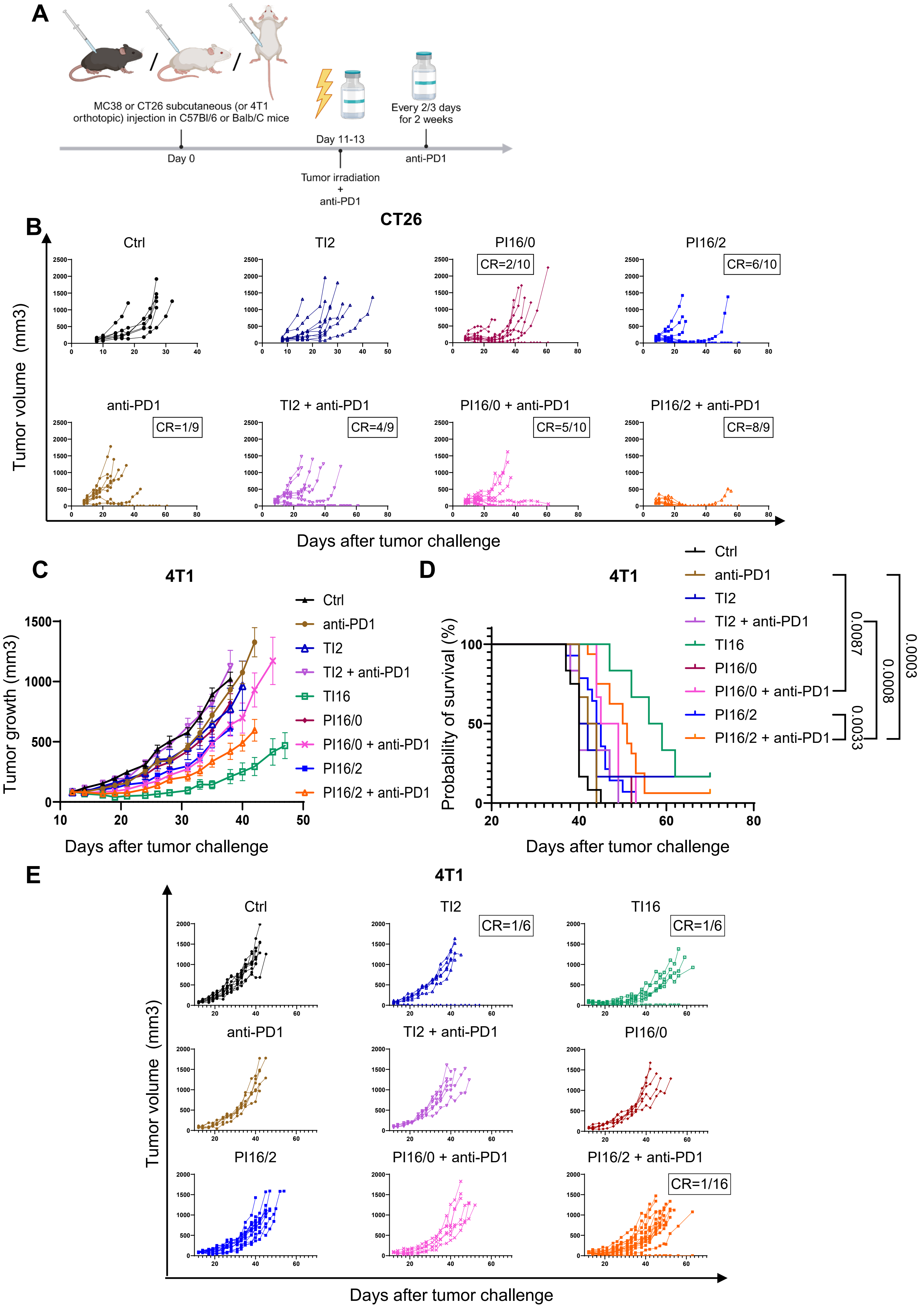

<u>Supplemental Figure 1</u>: Partial tumor irradiation combining LDRT and HDRT with anti-PD1 improves tumor control and survival in immunocompetent female mice bearing colorectal or breast tumors.

(**A**) Schematic diagram for the efficacy experiments performed on C57BL/6 or Balb/C mice with MC38 (subcutaneous), CT26 (subcutaneous), or 4T1 (orthotopic) cell lines and shown in Figure 2A-E, Supp. Figure 2B-E, Figure 6A-C, Supp. Figure 5C-E, Figure 8B-D, and Supp. Figure 6A-C. Created in BioRender. Mondini, M. (2024) BioRender.com/p56o265. (**B**) Individual curves of subcutaneous CT26 tumor growth in Balb/C mice from Figure 2D, E. CR= Complete Response. (**C**) Mean tumor volume growth of orthotopic 4T1 cells in Balb/C mice, starting on the day of irradiation (Ctrl n = 10, anti-PD1 n = 6, TI2 n = 6, TI2 + anti-PD1 n = 6, TI16 n = 6, PI16/0 n = 6, PI16/0 + anti-PD1 n = 6, PI16/2 n = 14, PI16/2 + anti-PD1 n = 16; combined from three independent experiments). Curves were stopped when the first mouse euthanasia occurred. Data were represented as the mean ± SEM. (**D**) Kaplan-Meier survival curves of the efficacy experiment on orthotopic 4T1 tumors shown in **C**. Numbers on the survival graph represent P values and were determined by Log-rank Mantel-Cox analysis. (**E**) Individual curves of orthotopic 4T1 tumor growth in Balb/C mice from the experiment shown in **C**-**D**. CR= Complete Response.

Supplemental Figure 2

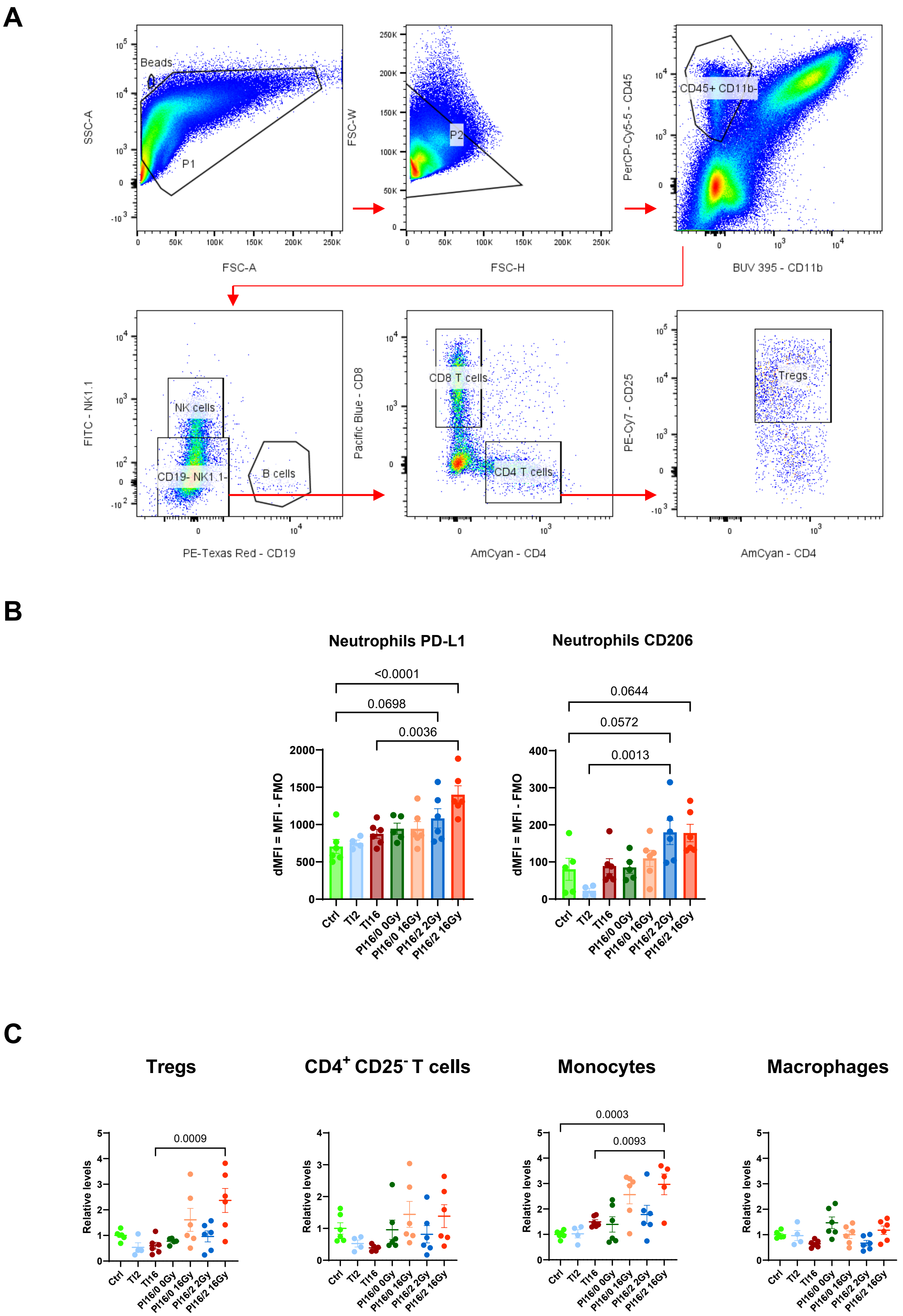

<u>Supplemental Figure 2</u>: Partial irradiation with LDRT and HDRT modulates the immune populations in the tumor microenvironment.

(**A**) Gating strategy associated with the lymphoid antibody panel used for the flow cytometry experiments shown in Figure 3B, Supp. Figure 2B-C, Supp. Figure 2C, Supp. Figure 3E and Figure 8A. The gating strategy used for myeloid cells is described in Gerbé de Thoré et al. [1]. (**B**) Difference between mean fluorescence intensity (MFI) and the fluorescence minus one (FMO) was calculated for the expression of PD-L1 and CD206 in the neutrophils identified in Figure 3B. Differentially-treated parts of partially-irradiated tumors were separated and considered as different groups. (**C**) Subcutaneous MC38 tumors from C57/Bl6 mice were treated and sampled as described in Figure 3A, and processed for flow cytometry 48 hours after treatment as described in Methods. Relative levels of the number of regulatory T cells (Tregs) (top left), $CD4^+$; $CD25^-$ T cells (bottom left), macrophages (top right), and monocytes (bottom right) per mg of tumor from the experiment shown in Figure 3B. Relative levels were calculated by comparing each individual value with the mean cell counts in Ctrl for each population. Differentially-treated parts of partially-irradiated tumors were separated and considered as different groups. Data were represented as the mean ± SEM, (Ctrl n = 6, TI2 n = 4, TI16 n = 6, PI16/0 0Gy n = 6, PI16/0 16Gy n = 6, PI16/2 2Gy n = 6, PI16/2 16Gy n = 6) (**B** and **C**). Outliers were identified using the ROUT test and excluded from the analysis. Numbers on these graphs represent P values and were determined by ordinary one-way ANOVA with the Šidák's multiple comparison test (**B** and **C**).

Supplemental Figure 3

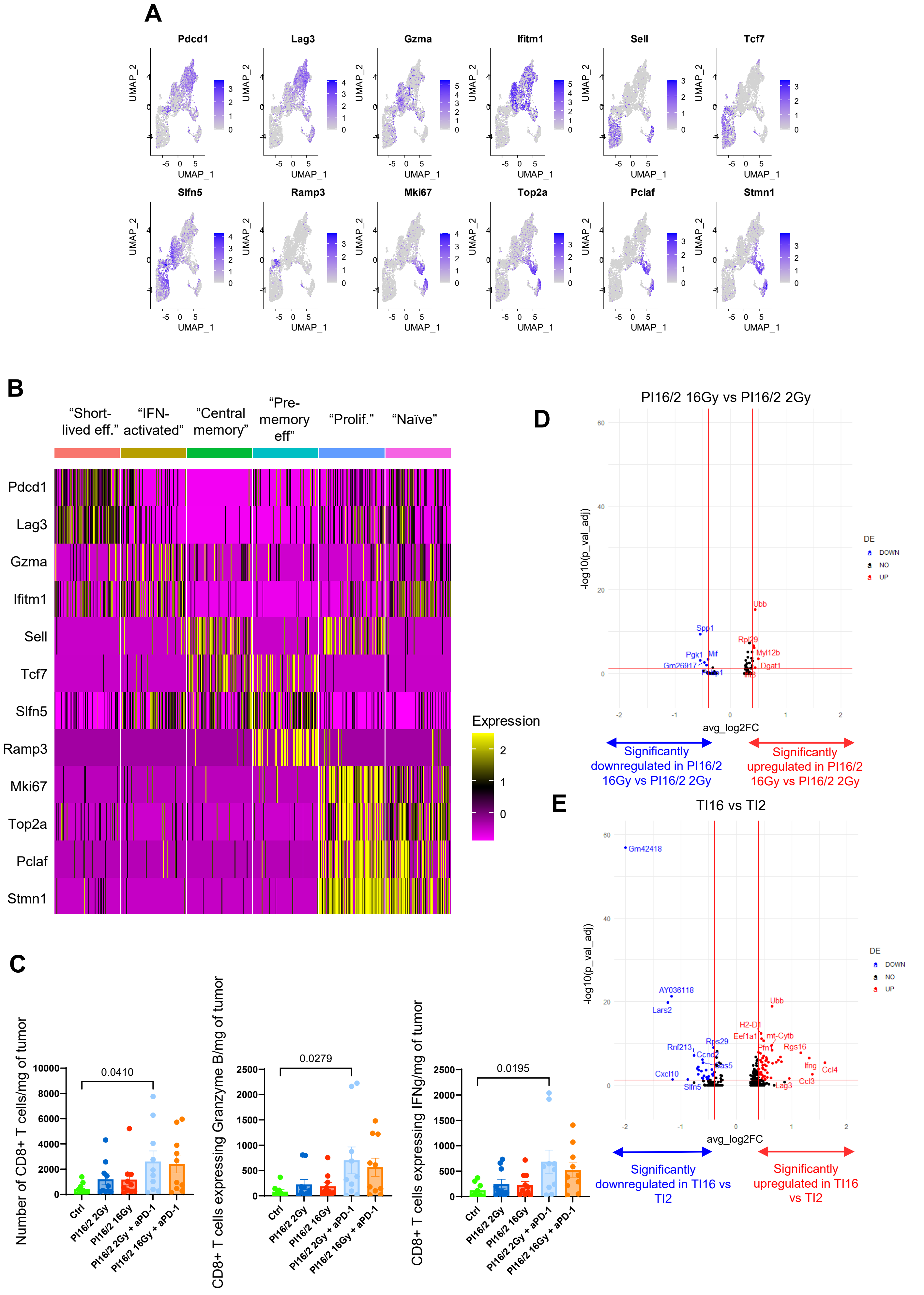

<u>Supplemental Figure 3</u>: Partial irradiation with LDRT and HDRT reshapes the phenotype of $CD8^+$ T cells within the tumor microenvironment.

(**A**) UMAP visualizations of key $CD8^+$ T cell mRNA expression at the single-cell level in CD8 T cells. These genes were used to identify the CD8 T cell subpopulations shown in Figure 4A. (**B**) Heatmap of selected DEGs displayed in **A** between the different $CD8^+$ T cell subpopulations (n=100 cells per subpopulation). (**C**) Subcutaneous MC38 tumors from C57/Bl6 mice were treated as described in Figure 3A, and received one inoculation of anti-PD1 (10 mg/kg, i.p.) on the day of irradiation (day 0). Tumors were sampled and processed for flow cytometry on day 2 after treatment as described in Methods and Figure 3A. The numbers of $CD8^+$ T cells (left), Granzyme B-expressing $CD8^+$ T cells (middle) and IFNgamma-expressing $CD8^+$ T cells (right) per mg of tumor were assessed. Differentially-treated parts of partially-irradiated tumors were separated and considered as different groups (Ctrl n = 9, PI16/2 2Gy n = 10, PI16/2 16Gy n = 10, PI16/2 2Gy + anti-PD1 n = 10, PI16/2 16Gy + anti-PD1 n = 10; combined from two independent experiments). Data are represented as the mean ± SEM, and n represents the number of mice/group. Numbers on these graphs represent P values and were determined by Kruskal-Wallis test with Dunn's multiple comparison test. (**D**) Volcano plot displaying DEGs between $CD8^+$ T cells from the portions PI16/2 16Gy and PI16/2 2Gy of PI16/2 tumors. Genes with > 0.4 log-fold changes (respectively <0.4) and an adjusted p value of 0.05 (based on Bonferroni correction) are highlighted in red (respectively in blue). Genes of interest labeled. (**E**) Volcano plot displaying DEGs between $CD8^+$ T cells from the TI16 and the TI2 tumors. Genes with > 0.4 log-fold changes (respectively <0.4) and an adjusted p value of 0.05 (based on Bonferroni correction) are highlighted in red (respectively in blue). Genes of interest are labeled.

Supplemental Figure 4

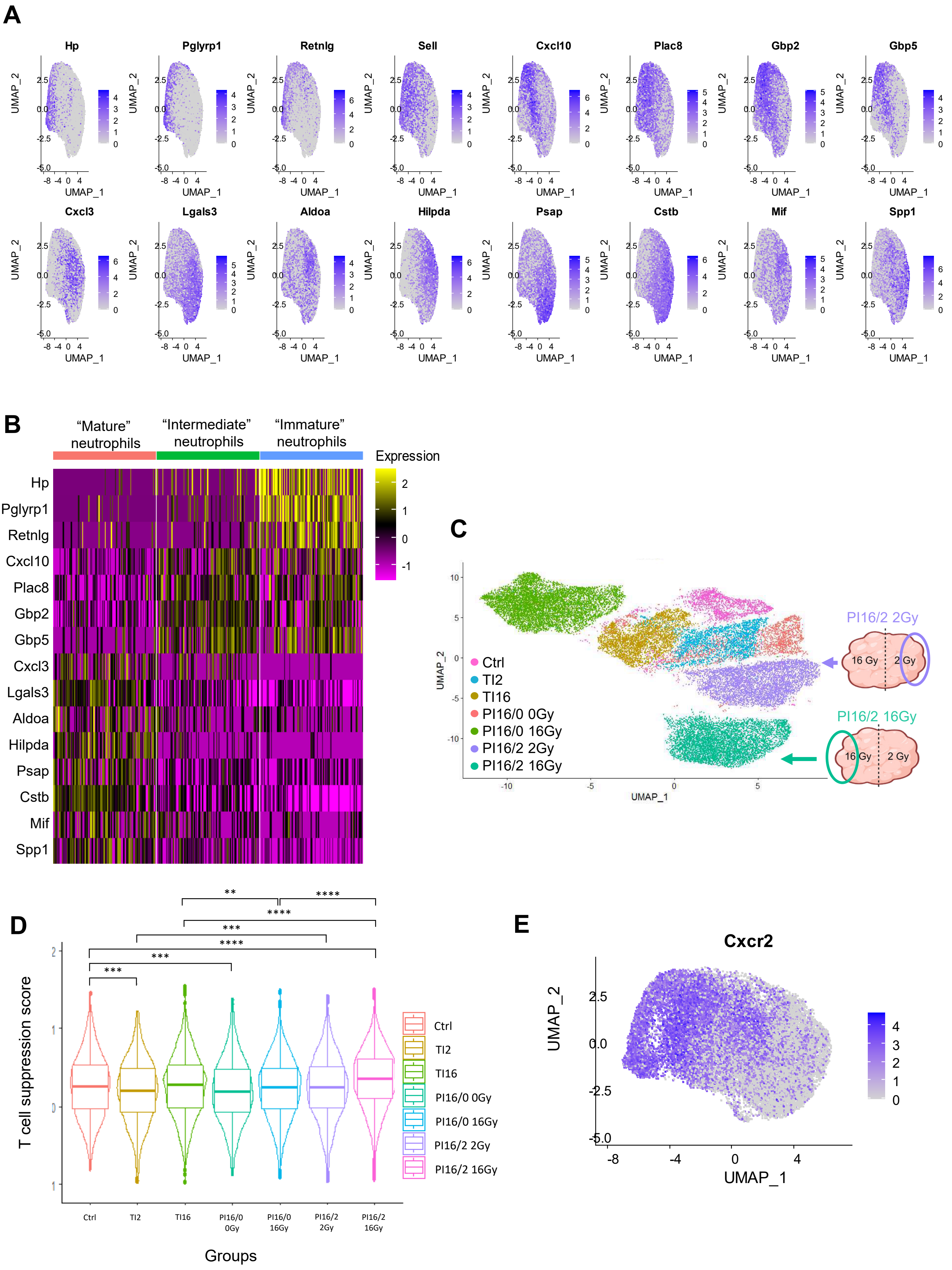

Supplemental Figure 4: Partial irradiation combining LDRT and HDRT reshapes the phenotype of neutrophils within the tumor microenvironment.

(**A**) UMAP projection of key neutrophil mRNA expression at the single-cell level in neutrophils. These genes were used to identify the neutrophil subpopulations shown in Figure 6A. (**B**) Heatmap of selected DEGs displayed in **A** between the different neutrophil subpopulations (n=100 cells per subpopulation). (**C**) Supervised dimension reduction visualization as a UMAP projection showing neutrophils isolated in a (n= 21,215 cells) and their projection according to their treatment condition. Both portions of PI16/2 are indicated with schematic tumors and colored arrows (purple for PI16/2 2Gy, green for PI16/2 16Gy). Schematic images created in BioRender. Mondini, M. (2024) BioRender.com/j74g766. (**D**) Expression score levels of the T cell suppression-associated gene signature in neutrophil populations depending on the treatment group. Each point represents the expression level of this feature (T cell suppression) at a single-cell level in the neutrophil population. The horizontal line drawn inside of the box represents the median. The lower and upper hinges represent the first and third quartiles, respectively. The upper whisker extends from the upper hinge to the largest value within 1.5 times the interquartile range (IQR) from the hinge. Similarly, the lower whisker extends from the lower hinge to the smallest value within 1.5 times the IQR from the hinge. Data beyond the end of the whiskers are outlying points and are plotted individually. Expression scores were generated using the AddModuleScore function. P values were determined by Mann-Whitney U test (**$P < 0.01$; ***$P < 0.001$; ****$P < 0.0001$). Exact P values are provided as a Source Data file. (**E**) UMAP projection of *CXCR2* mRNA expression at the single-cell level in neutrophils.

Supplemental Figure 5

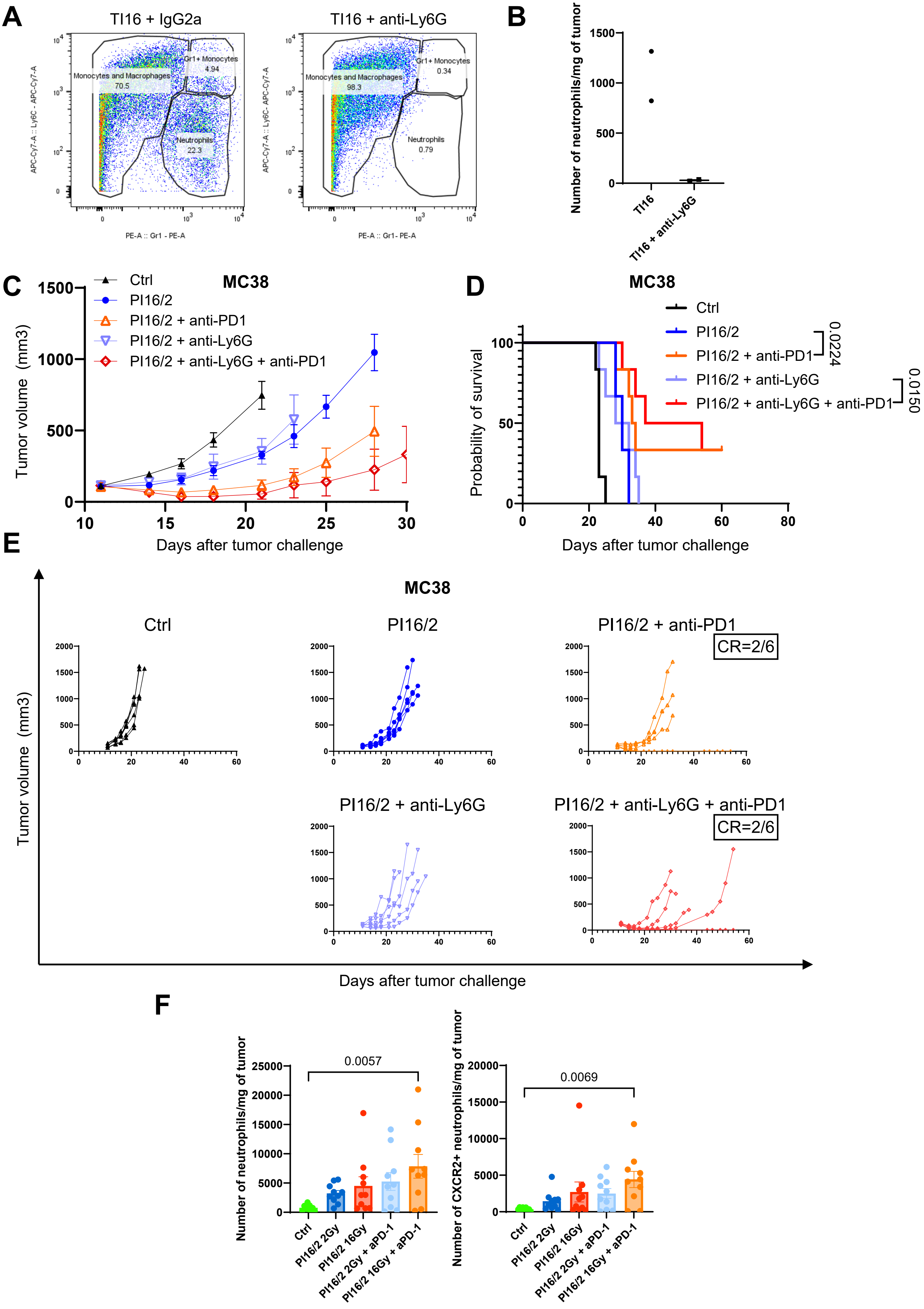

Supplemental Figure 5: Neutrophil depletion in tumors partially irradiated with LDRT and HDRT improves response to anti-PD1.

(**A**) Gating strategy for the detection of neutrophils in the flow cytometry experiment shown in Supp. Figure 5B. (**B**) Flow cytometry experiment analysis on neutrophils after inoculation with an anti-Ly6G. Subcutaneous MC38 tumors from C57BL/6 mice (*n*=2 mice per group) were treated and sampled as described in Figure 3A, and anti-Ly6G was administered on the day of irradiation. Data were represented in numbers of cells per mg of tumor. (**C**) Mean tumor volume growth of subcutaneous MC38 cells in C57/Bl6 mice, starting on the day of irradiation, with *n*=6 mice in all groups. Depletion of neutrophils with anti-Ly6G was performed as described in Methods. Curves are stopped as the first sacrifice occurs. Data were represented as the mean ± SEM, and *n* represents the number of mice/group. (**D**) Kaplan-Meier survival curves of the efficacy experiment shown in **C**. Numbers on the survival graph represent *P* values and were determined by Log-rank Mantel-Cox analysis. (**E**) Individual curves of subcutaneous MC38 tumor growth in C57BL/6 mice from the experiment shown in **C**. CR= Complete Response. (**F**) Subcutaneous MC38 tumors from C57/Bl6 mice were treated as described in Figure 3A, and received one inoculation of anti-PD1 (10 mg/kg, i.p.) on the day of irradiation (day 0). Tumors were sampled and processed for flow cytometry on day 2 after treatment as described in Methods and Figure 3A. The numbers of neutrophils (left) and $CXCR2^{+}$ neutrophils (right) per mg of tumor were assessed. Differentially-treated parts of partially-irradiated tumors were separated and considered as different groups (Ctrl *n* = 9, PI16/2 2Gy *n* = 10, PI16/2 16Gy *n* = 10, PI16/2 2Gy + anti-PD1 *n* = 10, PI16/2 16Gy + anti-PD1 *n* = 10; combined from two independent experiments). Data are represented as the mean ± SEM, and *n* represents the number of mice/group. Numbers on these graphs represent *P* values and were determined by ordinary one-way ANOVA with the Šidák's multiple comparison test.

Supplemental Figure 6

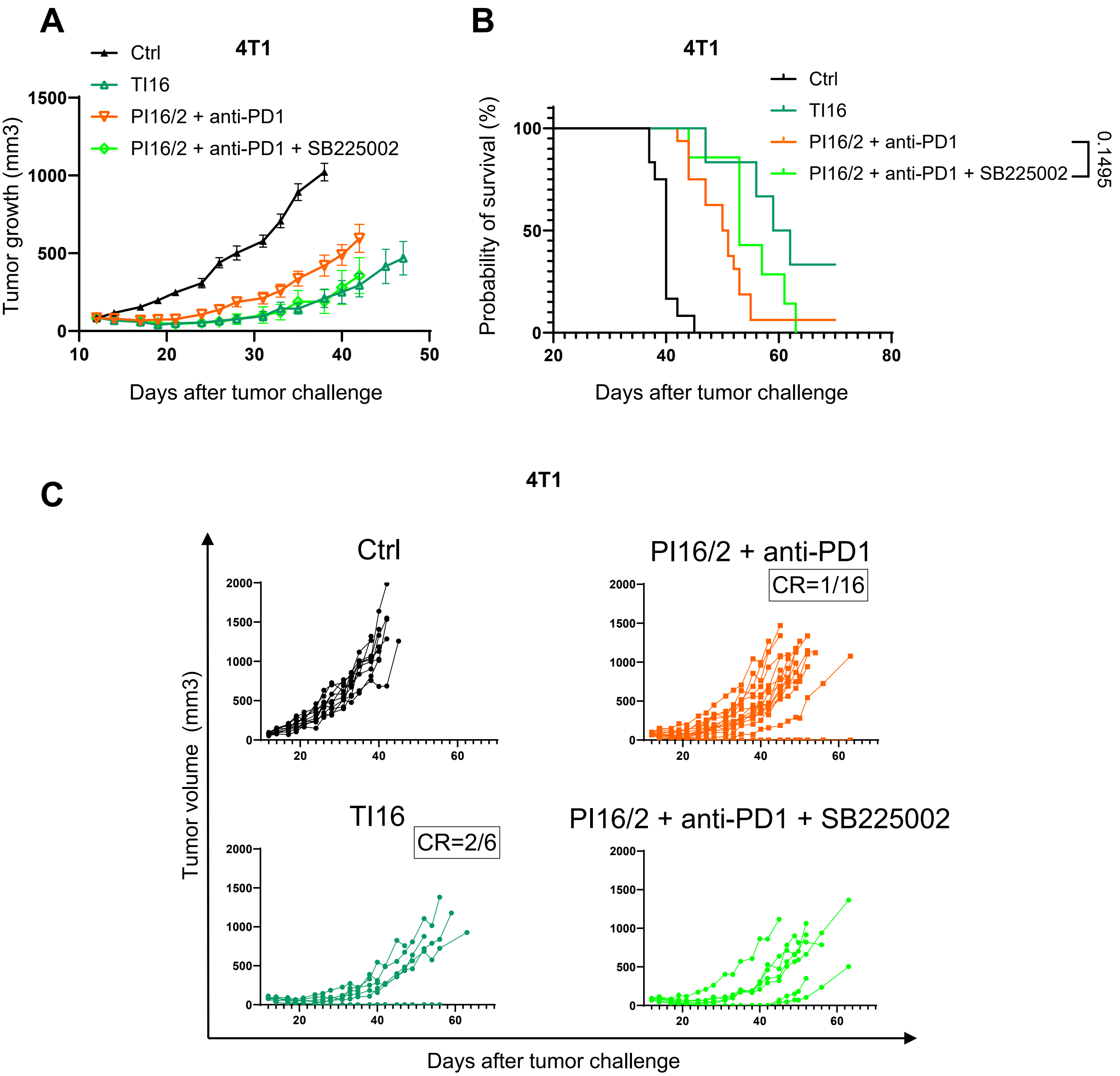

__Supplemental Figure 6__**: CXCR2 blockade in 4T1 tumors partially irradiated with LDRT and HDRT.**

(**A**) Mean tumor volume growth of orthotopic 4T1 cells in Balb/C mice, starting on the day of irradiation (Ctrl n = 12, TI16 n = 6, PI16/2 + anti-PD1 n = 16, PI16/2 + SB225002 + anti-PD1 n = 7; combined from three independent experiments). Curves are stopped as the first sacrifice occurs. Data were represented as the mean ± SEM, and n represents the number of mice/group. (**B**) Kaplan-Meier survival curves of the efficacy experiment shown in **A**. The number on the survival graph represents P values and was determined by Log-rank Mantel-Cox analysis. (**C**) Individual curves of orthotopic 4T1 tumor growth in Balb/C mice from the experiment shown in **A**. CR= Complete Response.

Supplemental Reference

1. De Thoré, M. G., Meziani, L., Deutsch, E. & Mondini, M. Chapter 2 - Cytofluorometric characterization of the myeloid compartment of irradiated mouse tumors. in *Methods in Cell Biology* (eds. Kraynak, J., Galluzzi, L., Marciscano, A. E. & Sato, A.) vol. 174 17–30 (Academic Press, 2023).
