# Supplementary material for: Non-homogenous intratumor ionizing radiation doses synergize with PD1 and CXCR2 blockade
Source: Nat Commun. 2024 Oct 14;15:8845. doi: 10.1038/s41467-024-53015-9 (PMC11471822; doi:10.1038/s41467-024-53015-9)
Supplement: Supplementary file 3 — Reporting Summary [file 41467_2024_53015_MOESM3_ESM.pdf]

nature portfolio

Corresponding author(s): Michele Mondini

Last updated by author(s): Sep 23, 2024

Reporting Summary

Nature Portfolio wishes to improve the reproducibility of the work that we publish. This form provides structure for consistency and transparency in reporting. For further information on Nature Portfolio policies, see our Editorial Policies and the Editorial Policy Checklist.

Statistics

For all statistical analyses, confirm that the following items are present in the figure legend, table legend, main text, or Methods section.

n/a	Confirmed	
☐	☒	The exact sample size (*n*) for each experimental group/condition, given as a discrete number and unit of measurement
☐	☒	A statement on whether measurements were taken from distinct samples or whether the same sample was measured repeatedly
☐	☒	The statistical test(s) used AND whether they are one- or two-sided<br>*Only common tests should be described solely by name; describe more complex techniques in the Methods section.*
☒	☐	A description of all covariates tested
☐	☒	A description of any assumptions or corrections, such as tests of normality and adjustment for multiple comparisons
☐	☒	A full description of the statistical parameters including central tendency (e.g. means) or other basic estimates (e.g. regression coefficient) AND variation (e.g. standard deviation) or associated estimates of uncertainty (e.g. confidence intervals)
☐	☒	For null hypothesis testing, the test statistic (e.g. *F*, *t*, *r*) with confidence intervals, effect sizes, degrees of freedom and *P* value noted<br>*Give P values as exact values whenever suitable.*
☒	☐	For Bayesian analysis, information on the choice of priors and Markov chain Monte Carlo settings
☒	☐	For hierarchical and complex designs, identification of the appropriate level for tests and full reporting of outcomes
☒	☐	Estimates of effect sizes (e.g. Cohen's *d*, Pearson's *r*), indicating how they were calculated

Our web collection on statistics for biologists contains articles on many of the points above.

Software and code

Policy information about availability of computer code

Data collection	BD FACSDiva software was used to acquire flow cytometry data<br>NovaSeq Control Software (Illumina) was used to acquire scRNAseq data
Data analysis	FlowJo v10 was used to analyze flow cytometry data.<br>GraphPad Prism v. 10 was used for statistical analyses.<br>Single-Cell RNA-Seq outputs were processed using the Cell Ranger software (10x Genomics). Downstream analysis was performed using the Seurat 4.0 and Monocle 3 packages in R.<br>The supervised UMAP was performed using the UWOT (version 0.1.11) R software package.<br>The PLS-DA study was performed using the package 'mixOmics' (version 6.18.1) in R software.<br>Custom code developed in R Studio is available from the Zenodo repository: https://doi.org/10.5281/zenodo.13684101

For manuscripts utilizing custom algorithms or software that are central to the research but not yet described in published literature, software must be made available to editors and reviewers. We strongly encourage code deposition in a community repository (e.g. GitHub). See the Nature Portfolio guidelines for submitting code & software for further information.

Data

Policy information about availability of data

All manuscripts must include a data availability statement. This statement should provide the following information, where applicable:

- Accession codes, unique identifiers, or web links for publicly available datasets
- A description of any restrictions on data availability
- For clinical datasets or third party data, please ensure that the statement adheres to our policy

The scRNA-seq datasets generated in this study are publicly available in the GEO repository GSE262699. All other data are reported in the source data file.

Research involving human participants, their data, or biological material

Policy information about studies with human participants or human data. See also policy information about sex, gender (identity/presentation), and sexual orientation and race, ethnicity and racism.

Reporting on sex and gender	N/A
Reporting on race, ethnicity, or other socially relevant groupings	N/A
Population characteristics	N/A
Recruitment	N/A
Ethics oversight	N/A

Note that full information on the approval of the study protocol must also be provided in the manuscript.

Field-specific reporting

Please select the one below that is the best fit for your research. If you are not sure, read the appropriate sections before making your selection.

☒ Life sciences ☐ Behavioural & social sciences ☐ Ecological, evolutionary & environmental sciences

For a reference copy of the document with all sections, see nature.com/documents/nr-reporting-summary-flat.pdf

Life sciences study design

All studies must disclose on these points even when the disclosure is negative.

Sample size	No statistical methods were used to predetermine sample size. For mouse studies, sample size was determined based on previous experience with mouse models.
Data exclusions	In Fig. 3B and Supplemental Fig. 2C,D outliers were identified using the ROUT test and excluded from the analysis, as detailed in the methods section. All data (including outliers) are reported as a Source Data file.
Replication	All experiments have been repeated at least twice, and all data are reported in the article, with the exception of data in Fig. 8A and Suppl. Fig. 6, which have been performed once.
Randomization	The day before the beginning of the treatments, mice were allocated to the different experimental groups according to their tumor size, to obtain homogeneous tumor volumes among groups.
Blinding	Investigators were not blinded during the study as they were also in charge of administrating the treatments

Reporting for specific materials, systems and methods

We require information from authors about some types of materials, experimental systems and methods used in many studies. Here, indicate whether each material, system or method listed is relevant to your study. If you are not sure if a list item applies to your research, read the appropriate section before selecting a response.

Materials & experimental systems

n/a	Involved in the study
☐	☒ Antibodies
☐	☒ Eukaryotic cell lines
☒	☐ Palaeontology and archaeology
☐	☒ Animals and other organisms
☒	☐ Clinical data
☒	☐ Dual use research of concern
☒	☐ Plants

Methods

n/a	Involved in the study
☒	☐ ChIP-seq
☐	☒ Flow cytometry
☒	☐ MRI-based neuroimaging

Antibodies

Antibodies used

The anti-PD1 (clone RMP1-14, BE0146, 10 mg/kg after SBRT and at 5 mg/kg, 3/week for 2 weeks), the rat IgG2a isotype control (clone 2A3, BE0089, 10 mg/kg after SBRT and at 5 mg/kg 3/week for 2 weeks) the anti-CD8 antibodies (clone 2.43, BE0061, 5 mg/kg) and anti-Ly6G (1clone 1A8, BE0075-1, 10 mg/kg), the rat IgG2b (clone LTF-2, BE0090, 5 mg/kg) and rat IgG2a isotype control (clone 2A3, BE0089, 10 mg/kg), were purchased from BioXcell.

For flow cytometry: Anti-IFNγ BV786 (clone XMG1.2, BD Horizon, 563773, 1/50), anti-Granzyme B PE-Cy7 (clone NGZB, eBioscience, 25-8898-80, 1/200), anti-CD69 APC (clone H1.2F3, BD Biosciences, 560689, 1/200), anti-CD11b BUV395 (clone M1/70, BD Biosciences, 563553, 1/400), anti-CD8a BV421 (clone 53-6.7, BD Biosciences, 563898, 1/400), anti-CD62L BUV737 (clone MEL-14, BD Biosciences, 612833, 1/200), anti-CD45 PerCP-Cy5.5 (clone 30-F11, BD Biosciences, 550994, 1/200), anti-Siglec F PE-CF594 (clone E50-2440, BD Biosciences, 562757, 1/100), anti-LAG3 BV650 (clone C9B7W, BioLegend, 125227, 1/50), anti-PD1 (CD279) BV605 (clone 29F.1A12, BioLegend, 135220, 1/100), anti-CD25 PE-Cy7 (clone PC61, BD Biosciences, 552880, 1/100), anti-CD4 BV510 (clone RM4-5, BioLegend, 100559, 1/400), anti-CD19 PE-CF594 (clone 1D3, BD Biosciences, 562291, 1/200), anti-NK1.1 FITC (clone PK136, BD Biosciences, 553164, 1/100), anti-Gr1 PE (clone RB6-8C5, ThermoFisher Scientific, 14-5931-82, 1/200), anti-CD182 (CXCR2) PE (clone 3F10-B3, BioLegend, 163903, 1/50), Anti-CD45 FITC (clone 30-F11, BioLegend, 103107, 1/200), anti-CD45 APC-Cy7 (clone REA737, Miltenyi Biotec, 130-110-662, 1/50), anti-Ly6G BV421 (clone 1A8, BD Biosciences, 562737, 1/400), anti-Ly6G PerCP Cy5.5 (clone REA526, Miltenyi Biotec, 130-117-500, 1/50), anti-I-A/I-E BV510 (clone 2G9, BD Biosciences, 743871, 1/200), anti-CD64 PE-Cy7 (clone X54-5/7.1, BioLegend, 139314, 1/200), anti-CD206 PerCP Cy5.5 (clone C068C2, BioLegend, 141716, 1/200), anti-CD274 BV650 (clone MIH5, BD Biosciences, 740614, 1/100), anti- Ly6C APC-Cy7 (clone AL-21, BD Bioscences, 560596, 1/100), anti- Ly6C AlexaFluor 700 (clone HK1.4, BioLegend, 128024, 1/50), anti-CD11c BV605 (clone N418, BioLegend, 117334, 1/100), anti-CD103 BV711 (clone 2E7, BioLegend, 121435, 1/100).

Validation

All antibodies have been validated by the vendor, and/or used in previous publications (e.g. DOI: 10.1016/bs.mcb.2022.08.004; DOI: 10.1136/jitc-2021-003519)

Eukaryotic cell lines

Policy information about cell lines and Sex and Gender in Research

Cell line source(s)

MC38 cell line originates from a chemically induced colon cancer in a female C57Bl/6 mouse (PMID: 1149045). MC38 cells were purchased from Kerafast.
CT26.WT is a clonal cell line generated from an N-nitroso-N-methylurethane-(NNMU) induced, undifferentiated colon carcinoma cell line from BALB/c mice.
4T1 is a 6-thioguanine resistant cell line selected from the 410.4 mammary carcinoma of a BALB/c female mouse.
CT26.WT and 4T1 tumor cells were purchased from ATCC.

Authentication

MC38 cells were authenticated by the supplier (authentication data available at https://www.kerafast.com/PDF/KF_STR_MC38_ENH204FP.pdf).
Cells were used to a maximum of 10 passages and no further authenticated in our laboratory.

Mycoplasma contamination

Mycoplasma contamination was assessed using the MycoAlert® Mycoplasma Detection Kit, giving negative results

Commonly misidentified lines
(See ICLAC register)

No commonly misidentified cell lines were used in this study

Animals and other research organisms

Policy information about studies involving animals; ARRIVE guidelines recommended for reporting animal research, and Sex and Gender in Research

Laboratory animals

Female mice aged 7-8 weeks of the C57BL/6 JRj or BALB/c JRj strains were purchased from Janvier CERT (Le Genest St. Isle, France)

Wild animals

The study does not involve wild animals

Reporting on sex

All animals used were female

Field-collected samples	The study did not involve samples collected from the field
Ethics oversight	Animal experiments were performed in compliance with French and European regulations on the protection of animals used for scientific purposes (EC Directive 2010/63/EU and French Decree 2013–118). All experiments were approved by the Ethics Committee CEEA26 of Gustave Roussy (approval number I-94-076-11) and #81 at IRSN (approval number E92-032-01) and authorized by the French Ministry of Research.

Note that full information on the approval of the study protocol must also be provided in the manuscript.

Plants

Seed stocks	*Report on the source of all seed stocks or other plant material used. If applicable, state the seed stock centre and catalogue number. If plant specimens were collected from the field, describe the collection location, date and sampling procedures.*
Novel plant genotypes	*Describe the methods by which all novel plant genotypes were produced. This includes those generated by transgenic approaches, gene editing, chemical/radiation-based mutagenesis and hybridization. For transgenic lines, describe the transformation method, the number of independent lines analyzed and the generation upon which experiments were performed. For gene-edited lines, describe the editor used, the endogenous sequence targeted for editing, the targeting guide RNA sequence (if applicable) and how the editor was applied.*
Authentication	*Describe any authentication procedures for each seed stock used or novel genotype generated. Describe any experiments used to assess the effect of a mutation and, where applicable, how potential secondary effects (e.g. second site T-DNA insertions, mosiacism, off-target gene editing) were examined.*

Flow Cytometry

Plots

Confirm that:

☒ The axis labels state the marker and fluorochrome used (e.g. CD4-FITC).

☒ The axis scales are clearly visible. Include numbers along axes only for bottom left plot of group (a 'group' is an analysis of identical markers).

☒ All plots are contour plots with outliers or pseudocolor plots.

☒ A numerical value for number of cells or percentage (with statistics) is provided.

Methodology

Sample preparation	C57BL/6 mice were euthanized, and MC38 tumors were collected as described in “Tumor sampling” section. Tumors were weighed, finely chopped, and dissociated with digestive enzyme (Tumor Dissociation Kit, mouse, Miltenyi Biotec, 130-096-730) according to the manufacturer’s instructions for 30 min at 37°C in the ThermoMixer® C (Eppendorf). Tumor digestions were then mechanically disrupted and filtered through a 40 µm nylon filtration cell strainer (Cell Strainer 40 µm Nylon, Falcon, USA, 352340).<br>Resulting single cells were incubated at 4°C with anti-CD16/32 (clone 93, BioLegend, 101319) antibodies for 10 min. The cells were stained using the same protocols as previously described (DOI: 10.1016/bs.mcb.2022.08.004). To analyze IFNγ and Granzyme-B intracellular levels, cells were stimulated for 2 hours at 37°C with PMA/ionomycin before incubation with anti-CD16/32 antibodies followed by membrane staining. Cells were then fixed using 4% paraformaldehyde for 15 min at 4°C and permeabilized using Perm/Wash Buffer (BD Perm/Wash) for intracellular cytokine staining.
Instrument	LSR Fortessa flow cytometer (BD)
Software	BD FACSDiva software
Cell population abundance	Cell sorting was not performed in this study
Gating strategy	For myeloid cells, the gating strategy is detailed in DOI: 10.1016/bs.mcb.2022.08.004<br>For lymphoid cells, the gating strategy is described in the Suppl. Fig. 2A

☒ Tick this box to confirm that a figure exemplifying the gating strategy is provided in the Supplementary Information.
